# Maintenance and generation of proton motive force are both essential for expression of phenotypic antibiotic tolerance in bacteria

**DOI:** 10.1128/spectrum.00832-23

**Published:** 2023-08-25

**Authors:** Yingkun Wan, Edward Wai Chi Chan, Sheng Chen

**Affiliations:** 1 Department of Infectious Diseases and Public Health, Jockey Club College of Veterinary Medicine and Life Sciences, City University of Hong Kong, Kowloon, Hong Kong; 2 Department of Applied Biology and Chemical Technology, State Key Lab of Chemical Biology and Drug Discovery, The Hong Kong Polytechnic University, Hung Hom, Kowloon, Hong Kong; 3 City University of Hong Kong Chendu Research Institute, Chengdu, China; 4 Shenzhen Key Lab for Food Biological Safety Control, Hong Kong PolyU Shenzhen Research Institute, Shenzhen, China; The University of North Carolina at Chapel Hill, Chapel Hill, North Carolina, USA

**Keywords:** antibiotic tolerance, proton motive force, active response

## Abstract

Bacterial antibiotic tolerance, a phenomenon first observed in 1944, is known to be responsible for both onset and exacerbation of recurrent and chronic bacterial infections. The development of antibiotic tolerance was previously thought to be due to a switch to physiological dormancy when bacteria encounter adverse growth conditions. Our recent laboratory findings, however, showed that a set of genes related to the maintenance of proton motive force (PMF) are up-regulated under starvation, indicating that the tolerant sub-population, which are commonly known as persisters, can actively maintain their tolerance phenotypes. In this study, we investigated the relative functional roles of proteins involved in the maintenance and active generation of PMF in mediating tolerance formation in bacteria and found that the PspA and RcsB proteins play a key role in PMF maintenance in persisters, as deletion of genes encoding these two proteins resulted in significantly lower tolerance levels. Consistently, expression of the OsmC and Bdm proteins, which is under regulation by RcsB, is required to maintain PMF and the antibiotic tolerance phenotypes. On the other hand, the NuoL, Ndh, AppC, CyoB, and NuoF proteins, which are electron transport chain (ETC) components, were also found to be actively expressed in persisters in order to generate PMF to support functioning of various tolerance mechanisms such as efflux activities. Our data show that active generation of PMF is even more important than the PMF maintenance functions of PspA and RcsB in the expression of antibiotic tolerance phenotypes in persisters. Assessment of double- and triple-gene knockout strains, in which the PMF maintenance genes and those encoding ETC components were simultaneously deleted, confirms that these two groups of genes are both required for the expression of antibiotic tolerance phenotypes and that a lack of these functions would result in complete PMF dissipation and accumulation of antibiotics in the intracellular compartment of persisters and eventually cell death. Products of these genes are, therefore, ideal targets for future development of anti-tolerance agents.

**IMPORTANCE:**

In this work, bacteria were found to undergo active generation and maintenance of proton motive force (PMF) under adverse conditions, such as starvation so as to support a range of physiological functions in order to survive under such conditions for a prolonged period. The ability to maintain a substantial level of PMF was found to be directly linked to that exhibiting phenotypic antibiotic tolerance under nutrient starvation or other adverse conditions. These findings infer that bacteria do not simply become physiologically dormant when they become antibiotic tolerant, instead they need to produce a wide range of proteins including those which help prevent PMF dissipation, such as PspA and RcsB, and the electron transport chain components, such as NuoL and Ndh, that actively generate PMF even during long-term starvation. As antibiotic tolerant sub-population is known to play a role in eliciting recurrent and chronic infections, especially among patients with a weakened immune system, the PMF maintenance mechanisms identified in this work are potential targets for the development of new strategies to control recurrent and chronic infections.

## INTRODUCTION

Bacterial tolerance to antibiotics is a decades-old phenomenon first observed in 1944 by Joseph Bigger, who found that bacteria always harbored a small population that could survive treatment by antibiotics, even at high drug concentration (1). Members of such antibiotic tolerant sub-population were also known as persisters (2). Apart from antibiotics, tolerance to various stresses was also observed (3 – 6). Subsequent research works that followed up on Bigger’s discovery showed that other unfavorable growth conditions could induce the formation of tolerance to multiple stresses, including the deleterious effects of various antimicrobial compounds. Importantly, nutrient starvation is known to induce bacterial tolerance to multiple antibiotics (7). Other studies showed that tolerance was also inducible by acids, bile salt, and heat stresses (8).

Cellular mechanisms underlying onset of stress tolerance in bacteria have been a serious research topic which only became increasingly important in the past few years when scientists realized that bacterial stress tolerance is closely linked to chronic and recurrent infections, especially among immunocompromised patients (9). Antibiotic tolerance is also known to predispose the development of antibiotic resistance by enabling bacteria to survive and undergo genetic changes or acquire exogenous genetic elements under adverse conditions (10). Despite much research, however, there is no consensus on the key mechanism that gives rise to tolerance formation in bacteria. Physiological dormancy or shutting down of metabolic pathways when growth conditions are not favorable is generally regarded as the main factor that elicits onset of stress tolerance (11 – 13). It is believed that the vast majority of protein synthesis and physiological activities in the antibiotic tolerant sub-population have shut down completely under conditions that cause a large proportion of the population to switch to the stress-tolerant mode, such as nutrient starvation. Various signaling and physiological regulatory systems, including the toxin-antitoxin system, have been postulated to play a role in mediating the expression of stress tolerance phenotypes. A number of putative “tolerance” genes have also been identified (14). However, none of the tolerance mechanisms identified in previous studies could be defined as a major mechanism, as deletion of genes that encode these putative mechanisms fails to prevent the development of stress tolerance in bacteria. Based on these observations, researchers hypothesize that a vast and redundant tolerance induction network that involves both active and passive shutdown of physiological activities is responsible for tolerance formation and that it is almost impossible to prevent the development of tolerance by inhibiting a specific mechanism. We disputed this concept completely in a recent study by proving that bacteria actually need to actively maintain a tolerance phenotype in the long term and that inhibiting the ability to actively maintain the tolerance phenotype can eradicate antibiotic tolerant persisters completely (15). It should be noted that most of the previous studies on bacterial tolerance did not assess the level of stress tolerance for more than 24 h. In a starvation-induced antibiotic tolerance model, we found that a switch to physiological dormancy can only confer tolerance to antibiotics for approximately 48 h; after this period, bacteria need to maintain the transmembrane proton motive force (PMF) in order to remain being tolerant to antibiotics; without the ability to maintain a substantial level of PMF, the persisters died gradually, and the entire tolerant population disappeared within 7 days. Based on this finding, we further proposed the concept of “tolerance maintenance” and identified some of the mechanisms concerned. We found that the PspA protein was responsible for maintaining the PMF during nutrient starvation and that the persisters still actively generate PMF by undergoing a certain level of oxidative phosphorylation, even after they have encountered complete nutrient starvation for 24 h. In this previous work, we showed that *pspA* was one of the most up-regulated genes upon onset of starvation-induced tolerance. The Psp response was found to play a role in regulating the pathogenicity of enterobacteria (16). The Psp proteins were also previously found to be responsible for maintaining PMF under PMF-dissipating conditions (17), but this function has not been reported as being important for tolerance formation. Consistently, we also identified a number of membrane-bound transporters that become functionally active during starvation and play a role in the formation of stress tolerance; among them, TolC-EmrKY and ChaAB are two major efflux systems that can exude antibiotics or various metabolites out of the bacterial cell during starvation, enabling bacteria to survive in extreme environment (18). If PMF is important for tolerance development, we postulate that bacteria do not employ PspA as the only PMF maintenance protein but utilize a range of mechanisms to safeguard a sufficient level of PMF to maintain survival fitness under adverse growth conditions. In the literature, the Rcs regulon is another regulatory system documented to be responsible for maintaining PMF. The *rcs* operon, which contains a total of 19 genes, encodes the production of the colanic acid-rich capsular polysaccharide (19). Colonic acid was found to help maintain PMF when the bacterial cell encounters specific stress, but this compound has not been postulated to play a role in tolerance formation (20). In this study, we tested whether specific genes in this operon were also responsible for PMF maintenance during starvation. The test genes include *osmC* and *osmB*, which encode a periplasmic peroxidase and a lipoprotein, respectively. We hypothesize that the products of these two genes may play a role in mediating functional linkage between the RpoS and Rcs regulon and presumably contribute to maintenance of PMF (21). Another gene tested in this work is *bdm*, the expression of which is regulated by RcsB. This gene was reported to be involved in promoting flagellum synthesis in *Escherichia coli* (22). It is not clear whether this gene is involved in PMF maintenance.

In our previous work, we showed that PMF maintenance functions were not only limited to preventing dissipation of PMF but also involved active generation of PMF by respiratory activities. The electron transport chain (ETC) is responsible for the generation of ATP and PMF, but the activities of electron transport chain components when bacteria encounter prolonged starvation and develop antibiotic tolerance have not been characterized. Our preliminary data showed that the enzymes NADH dehydrogenase I and NADH dehydrogenase II, which are the key components of the electron transport chain encoded by the genes *nuoL* and *ndh* (23, 24), also play a key role in mediating tolerance formation (15). In this work, we further tested the functional role of other components of the electron transport chain in generating PMF and, hence, their role in maintaining the tolerance phenotype. We found that deletion of genes encoding the electron transport chain components results in unprecedented and rapid death of persisters, which is never achievable by deleting a single putative tolerance gene. Therefore, we confirm that sustainable generation of PMF and preventing dissipation of PMF are both important in maintaining a tolerance phenotype. These findings provide important insight into the development of effective strategies to eradicate antibiotic-tolerant persisters and prevent occurrence of chronic and recurrent infections.

## MATERIALS AND METHODS

### Materials

In this work, *E. coli* strain BW25113 (25) was used as a wild-type (WT) control strain, from which specific gene knockout strains were created for functional studies. LB broth and LB agar were used in all bacterial culture, except that MH broth (Hopebio Company) was used in MIC tests. Saline of 0.85% was used to create a starvation-induced antibiotic-tolerant population. L-Arabinose (Sigma) was used in preparation of competent cells. All antibiotics used in this study were purchased from SIGMA. All single-knockout strains were obtained from the Keio Collection.

### Starvation-induced tolerance assay

Bacterial population was subjected to starvation by using the following approach. Bacterial culture at mid-log phase was subjected to centrifugation (6,000 *g*, 5 min), followed by removal of the supernatant and re-suspension of the pellet in 0.85% saline. Antibiotic (100 µg/mL) was added to the cell suspension and then every second day for up to 1 month unless stated otherwise. CCCP (10 µM), an ionophore known to cause PMF dissipation, was included as control in the assessment of effect of deletion of specific genes. During this period, the bacterial suspension was continually incubated at 37°C with shaking at 250 rpm. The survival rate of the bacteria was determined by recording the CFU (colony-forming unit) in the bacterial suspension daily. Every assay was repeated three times, and error bars were displayed in the graphs.

### Creation of double-gene knockout strains

The plasmid pKD3, which carries the selectable antibiotic chloramphenicol resistance gene (*cat*) flanked by FRT (FLP recognition target) sites, was used in a double-gene knockout experiment, in which the gene to be deleted was replaced by *cat* gene (26). In brief, competent cells of a single-gene knockout strain were prepared by inoculating an overnight culture of strain BW25113 and incubating at 37°C until OD_600_ reached 0.3–0.5, followed by washing for three times with 10% glycerol at 4°C. The plasmid pKD46, which is a temperature-sensitive plasmid expressing the λ Red recombinase protein, was transformed into competent cells, followed by a selection of ampicillin-resistant transformants on agar plates containing 100 µg/mL ampicillin. In the next step, competent cells of strains carrying pKD46 were prepared by incubating the strains at 30°C until OD_600_ reached 0.3–0.4, followed by the addition of 0.5% arabinose, incubation for 1 h, and washing in glycerol for three times at 4°C. Finally, the homologous sequence-FRT-flanked *cat* gene was transformed into the competent cells. The double-gene knockout strains were selected on agar plates containing 50 µg/mL chloramphenicol.

### Membrane potential assay

In order to investigate whether the Psp response and Rcs response are both responsible for maintaining PMF while ETC genes can generate new PMF in bacteria which have encountered nutrient starvation for 24 h, the membrane potential of the PMF maintenance and ETC gene knockout strains and a series of double- and triple-knockout mutants, in which different combinations of these genes were deleted, were measured. DiSC3(5) was used as the fluorescent dye for the measurement of PMF-dependent uptake functions, and the excitation wavelengths and emission wavelength were 622 nm and 670 nm, respectively; KCl was used to provide anions in the assay. Valinomycin, which can completely disrupt the transmembrane PMF, was included as control. In detail, after adjusting the bacteria concentration to OD0.2, 1 µM DiSC3(5) and 100 mM KCl were first added to bacteria, and after 15 min treatment, 150 μL bacteria were spread to 96-well cell culture plate and then 5 µg/mL valinomycin was added to the positive control group.

### Fluorescence microscopy

An inverted microscope was used for visualization of the structural changes of antibiotic-tolerant persisters during various types of treatment, using laser of different wavelengths. For testing accumulation of Bocillin FL, the excitation wavelengths and emission wavelength were 488 ± 10 nm and 512 ± 10 nm, respectively. For assessment of accumulation of the Texas red gentamicin conjugate, the excitation wavelengths and emission wavelength were adjusted to 500–600 nm and 610 nm, respectively. The EMCCD camera was used to record the fluorescence signal. The images were analyzed by the ImageJ software (Fiji). Antibiotic accumulation in the transporter gene knockout strains which had been subjected to nutrient starvation for 6 days was observed under the fluorescence microscope upon treatment with Bocillin FL or the Texas red gentamicin conjugate. Briefly, 20 µg/mL Bocillin FL penicillin or 1.25 µg/mL Texas red gentamicin conjugate was added to the bacterial suspension, followed by treatment with the corresponding antibiotic for 30 min. 0.85% saline was agents to provide a clear background for fluorescence microscopy and then used to wash out the test. The fluorescence intensity in different samples was recorded and compared.

## RESULTS

### Up-regulated genes in persisters upon starvation for 24 h

**Fig 1:**
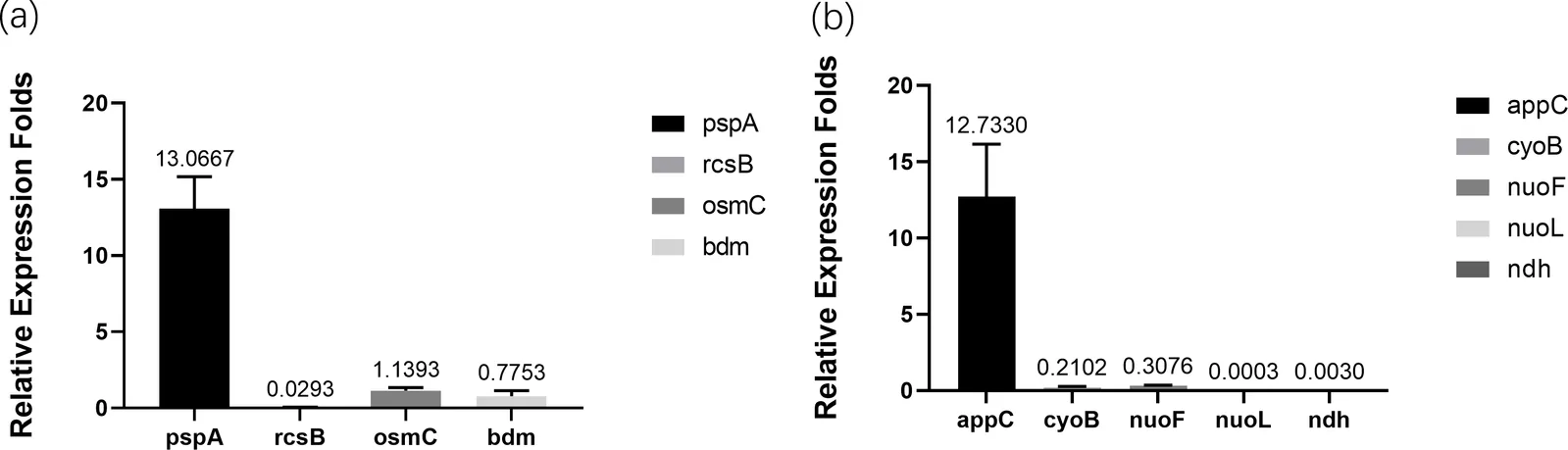
Expression levels of genes involved in active tolerance response. Relative expression levels of the genes *pspA*, *rcsB*, *osmC*, and *bdm* (**a**) and *appC*, *cyoB*, *nuoF*, *nuoL,* and *ndh* (**b**) in persisters of *E. coli* Bw25113 upon encountering starvation for 24 h, with log phase cells as control.

Our previous study showed that the phage shock protein PspA was over-expressed in bacteria which have encountered starvation for 24 h and that the protein contributed to antibiotic tolerance formation by maintaining the transmembrane PMF (15). In this study, we further explored the possibility that the *rcs* operon also plays a role in maintaining PMF. We also hypothesize that simply maintaining preexisting PMF is not sufficient for supporting the survival of persisters for a prolonged period and that active generation of PMF is required for that purpose; therefore, we also tested whether the ETC components were also involved in active generation of PMF and compared the relative functional role of the PMF maintenance and active PMF generation systems in enabling persisters to survive for a prolonged period. Figure 1 showed that, after encountering starvation for 24 h, the expression levels of representative genes in these two functional groups, namely, *pspA* in the PMF maintenance group and *appC* in ETC, are both up-regulated up to 12 times when compared to the log phase cells. Not surprisingly, expression levels of several genes, such as *rcsB* and *nuoL* in the PMF maintenance group and ETC, in cells under starvation stress were lower than that of the exponentially growing cells. Nevertheless, the observation that a number of genes in these two groups are expressed at a level higher than that of the log phase cells indicates that members of both of these two groups may play an essential role in tolerance formation and maintenance.

### Effect of deletion of various test genes in PMF maintenance

**Fig 2:**
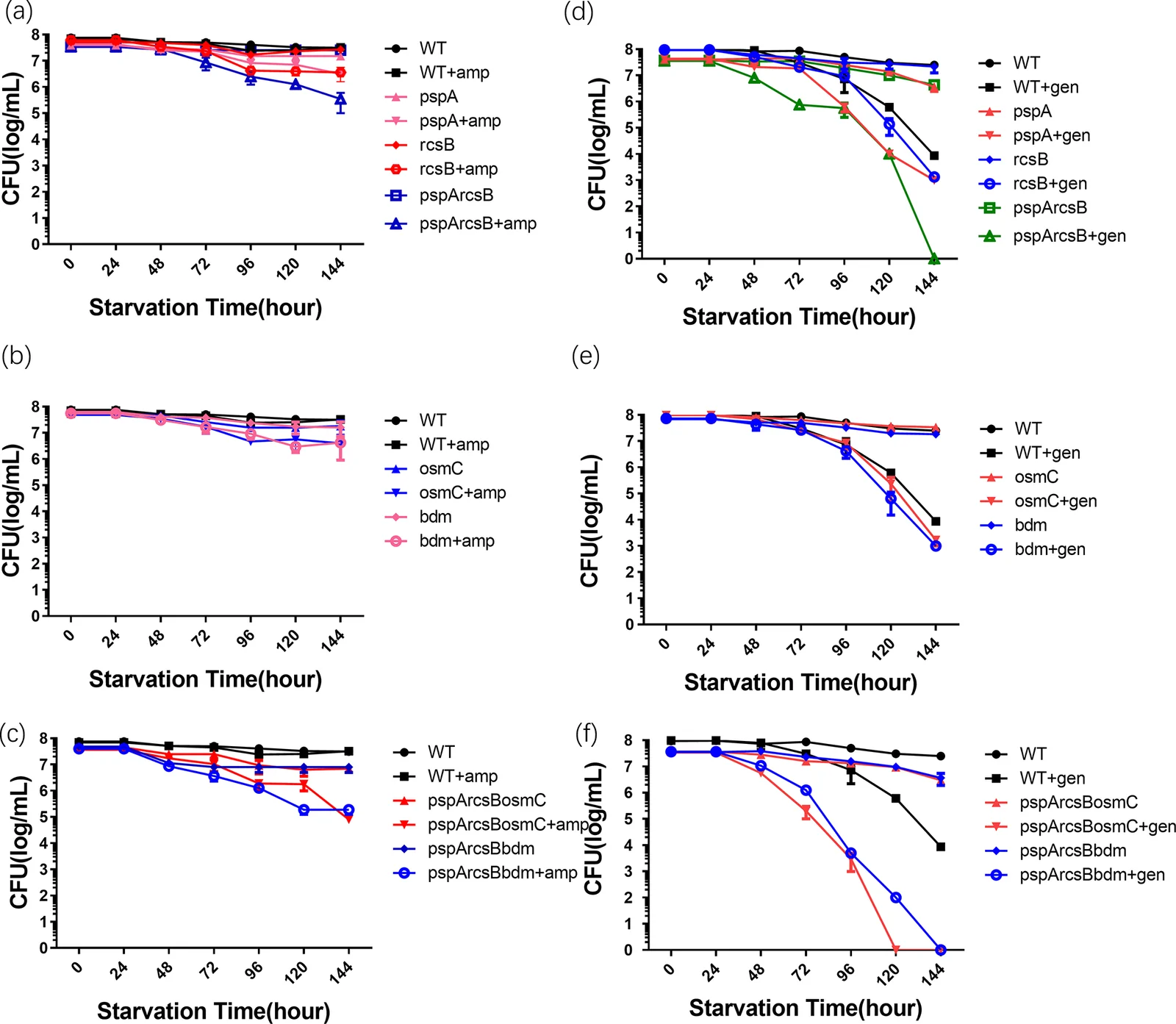
Tolerance level of strains in which PMF maintenance genes were deleted during a 6-day starvation period. Tolerance level of *pspA, rcsB, and pspArcsB* (**a**); *osmC* and *bdm* (**b**); and *pspArcsBosmC* and *pspArcsBbdm* (**c**) knockout strains with or without 100 µg/mL ampicillin treatment. Tolerance level of *pspA, rcsB,* and *pspArcsB* (**d**); *osmC* and *bdm* (**e**); and *pspArcsBosmC* and *pspArcsBbdm* (**f**) knockout strains with or without 10 µg/mL gentamicin treatment. *E. coli* strain. BW25113 was included as wild-type control.

We next tested the effect of deletion of putative PMF maintenance gene. The level of ampicillin tolerance of the *rcsB* and *osmC* gene knockout strains was found to decrease from 10^8^ to 10^7^ cells/mL during a 6- day starvation period, which was the same as that of the positive control, namely, the *pspA* knockout strain. Therefore, these three genes were regarded to be involved in tolerance formation (Fig. 2a and b). Double- and triple-knockout strains were then created to test the effects of simultaneous deletion of two or three of these genes on the degree of tolerance ampicillin treatment. Deletion of both *pspA* and *rcsB* resulted in a decrease in tolerance level from 10^8^ to 10^6^/mL, which was about 10 times that of the single-knockout strains observed during ampicillin treatment. It should be noted that the *pspAosmC* and *pspAbdm* double-knockout strains exhibited a tolerance level almost identical to the single-knockout strains, with the number of persisters decreasing from 10^8^ to 10^7^/mL only (Fig. S1). However, further deletion of *osmC* or *bdm* from the background of the *pspArcsB* knockout mutant eventually caused the persisters to die much more rapidly upon exposure to ampicillin, as the population size of persisters of the *pspArcsBosmC* and *pspArcsBbdm* triple-gene deletion mutant decreased from 10^8^ to 10^5^/mL during ampicillin treatment (Fig. 2c). This finding is consistent with the observation in the gene expression experiment in that the expression levels of *bdm* and *osmC* in cells under starvation were equal to or higher than that of the log phase cells (Fig. 1). The trend of reduction in the population size of persisters observed during gentamicin treatment was consistent with that of ampicillin treatment, except that the degree of reduction was more obvious. The tolerance level of the *pspA*, *rcsB*, *osmC,* and *bdm* single-knockout strains was found to decrease to 10^3^/mL eventually, whereas the persisters of the *pspArcsB* double-knockout strain were completely eradicated on day 6 (Fig. 2d and e). The triple-knockout strain *pspArcsBbdm* persisters were also eradicated on day 6, whereas the *pspArcsBosmC* persisters were completely eradicated on day 5 upon treatment with gentamicin (Fig. 2f). To confirm that the reduced tolerance levels of the gene knockout strains were not due to reduced susceptibility of the test antibiotics as a result of altered membrane structure, we tested the minimal inhibitory concentrations of ampicillin and gentamicin on these strains and compared to the wild-type strains. The results showed that the MICs of all the gene knockout strains were the same as that of the wild type, indicating that the reduced tolerance level was not related to reduced antibiotic susceptibility of the gene knockout strains. These findings confirm that the genes *pspA*, *rcsB*, *osmC,* and *bdm* are all involved in tolerance formation and that they act synergistically to maintain the tolerance phenotype, presumably by maintaining PMF. On the other hand, the ciprofloxacin treatment experiment showed that all gene knockout strains were eradicated at the same rate as that of the wild-type strain, with the size of the persister population decreasing from 10^8^/mL to 0/mL at the end of the 6-day starvation period (Fig. S2). It appears that even the persisters of the wild-type strain are already highly sensitive to ciprofloxacin and that abolishment of PMF maintenance functions does not result in more rapid drop in tolerance level.

### Effect of deletion of genes that encode electron transport chain components

**Fig 3:**
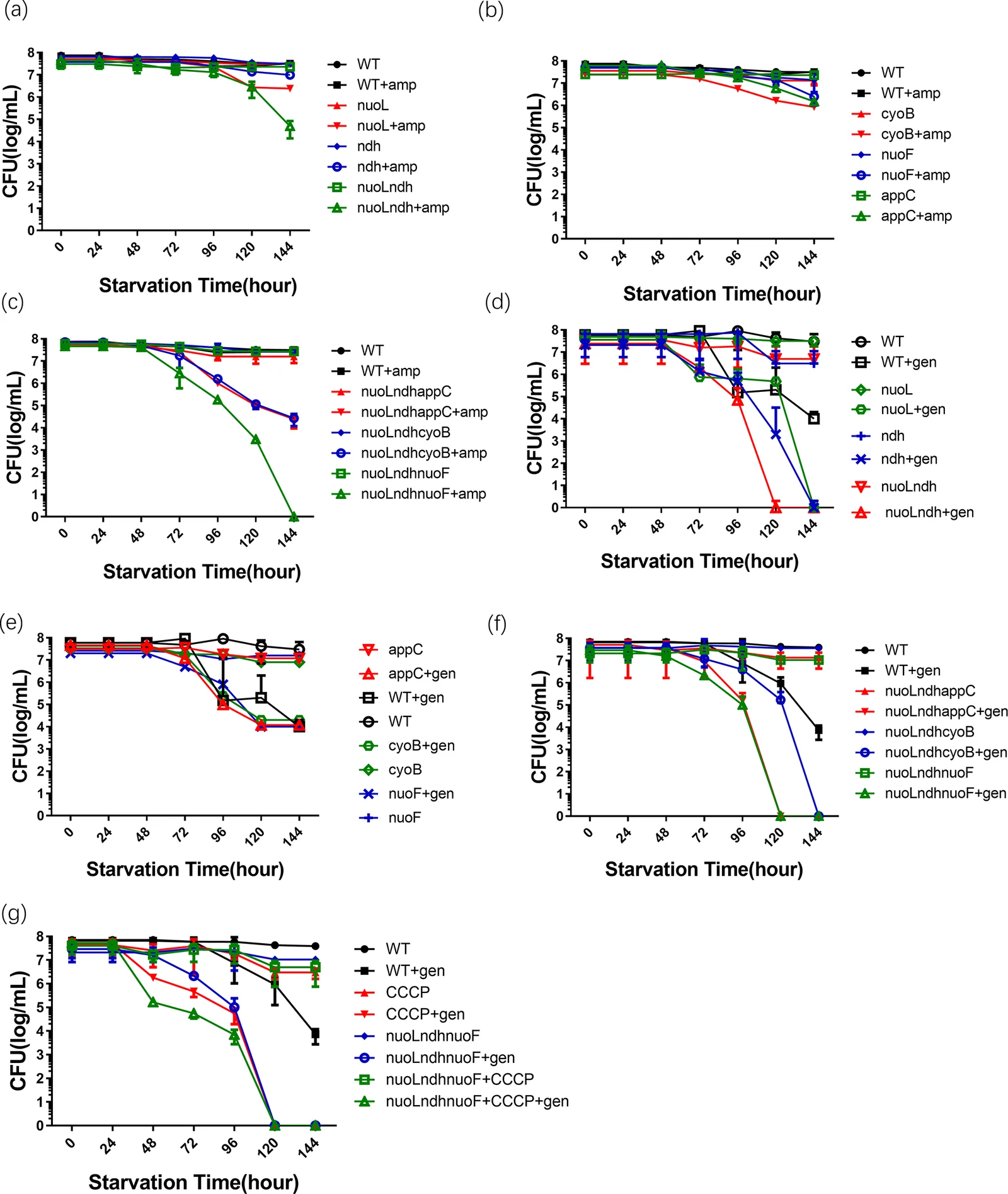
Changes in the level of tolerance of ETC gene knockout strains to ampicillin and gentamicin during the course of a 6-day starvation experiment. Tolerance level of *nuoL, ndh,* and *nuoLndh* (**a**); *cyoB, appC,* and *nuoF* (**b**); and *nuoLndhappC, nuoLndhcyoB,* and *nuoLndhnuoF* (**c**) knockout strains with or without treatment by 100 µg/mL ampicillin. Tolerance level of *nuoL, ndh,* and *nuoLndh* (**e**); *cyoB, appC,* and *nuoF* (**f**); and *nuoLndhappC, nuoLndhcyoB,* and *nuoLndhnuoF* (**g**) knockout strains with or without treatment by 10 µg/mL gentamicin. Tolerance level of *nuoLndhnuoF* (**g**) knockout strains with or without treatment with 10 µg/mL gentamicin and/or 10 µM CCCP. BW25113 was included as wild-type control.

We then tested the effect of deletion of the ETC gene on the antibiotic tolerance level of persister; surprisingly, we found that the impact of abolishment of ETC activities on survival of persisters was even more remarkable than that of deletion of PMF maintenance genes. First, our results showed that deletion of each of the *ndh* or *nuoF* gene could only reduce the population size of persisters to 10^7/^mL upon ampicillin treatment and that deletion of the *nuoL*, *appC,* and *cyoB* gene caused a reduction of the population size of persisters to 10^6^/mL (Fig. 3a and b). However, the population size of persisters of the *nuoL* and *ndh* double-knockout mutant was found to drop to 10^4^/mL despite the fact that the expression levels of these two genes in the persisters were significantly lower than that of the log phase cells (Fig. 1 and 3a). It should also be noted that, although the *appC* gene was over-expressed during starvation, deletion of this gene alone did not result in a significantly lower tolerance level when compared to the effect of deletion of the *nuoL* or *ndh* gene. Interestingly, although the other double-knockout strains did not exhibit such a dramatic reduction in tolerance level (Fig. S2), the persisters of the *nuoLndhnuoF* triple-knockout strain could be totally eradicated by ampicillin on day 6 of the experiment. Again, the other two triple-knockout strains remained fairly tolerant throughout the experiment (Fig. 3c). On the other hand, gentamicin also caused a significant decline in the population size of the persisters of the ETC gene knockout strains. Persisters of the *nuoL* and *ndh* gene knockout strains (Fig. 3d) were eradicated by gentamicin upon starvation for 6 days, whereas the tolerance level of the other three single-gene deletion mutants was almost the same as that of the wild type. It should be noted that the peristers of the *nuoLndh*, *nuoLndhnuoF,* and *nuoLndhappC* gene deletion mutants (Fig. 3f) could be completely eradicated by day 5. Since simultaneous deletion of the *nuoL*, *ndh*, and *nuoF* genes resulted in the largest degree of reduction in tolerance level, especially during treatment with gentamicin, we compared the effects of deletion of these genes on gentamicin tolerance with that of treatment with a non-lethal concentration of CCCP (Fig. 3g), an ionophore known to cause dissipation of PMF. The results showed that the level of reduction in tolerance level of CCCP-treated cells recorded upon gentamicin treatment was similar to that of the *nuoLndhnuoF* knockout strain, indicating that deletion of these three genes exhibited a PMF-dissipating effect similar to that conferred by CCCP. Unlike the different killing effects of ampicillin and gentamicin observed in different gene deletion mutants, all mutants exhibited the same killing rate as that of the wild-type strain during ciprofloxacin treatment (Fig. S2). In other words, deletion of the ETC genes did not result in a further reduction in tolerance level during ciprofloxacin treatment.

### Effect of simultaneous deletion of the ETC genes and PMF maintenance genes

**Fig 4:**
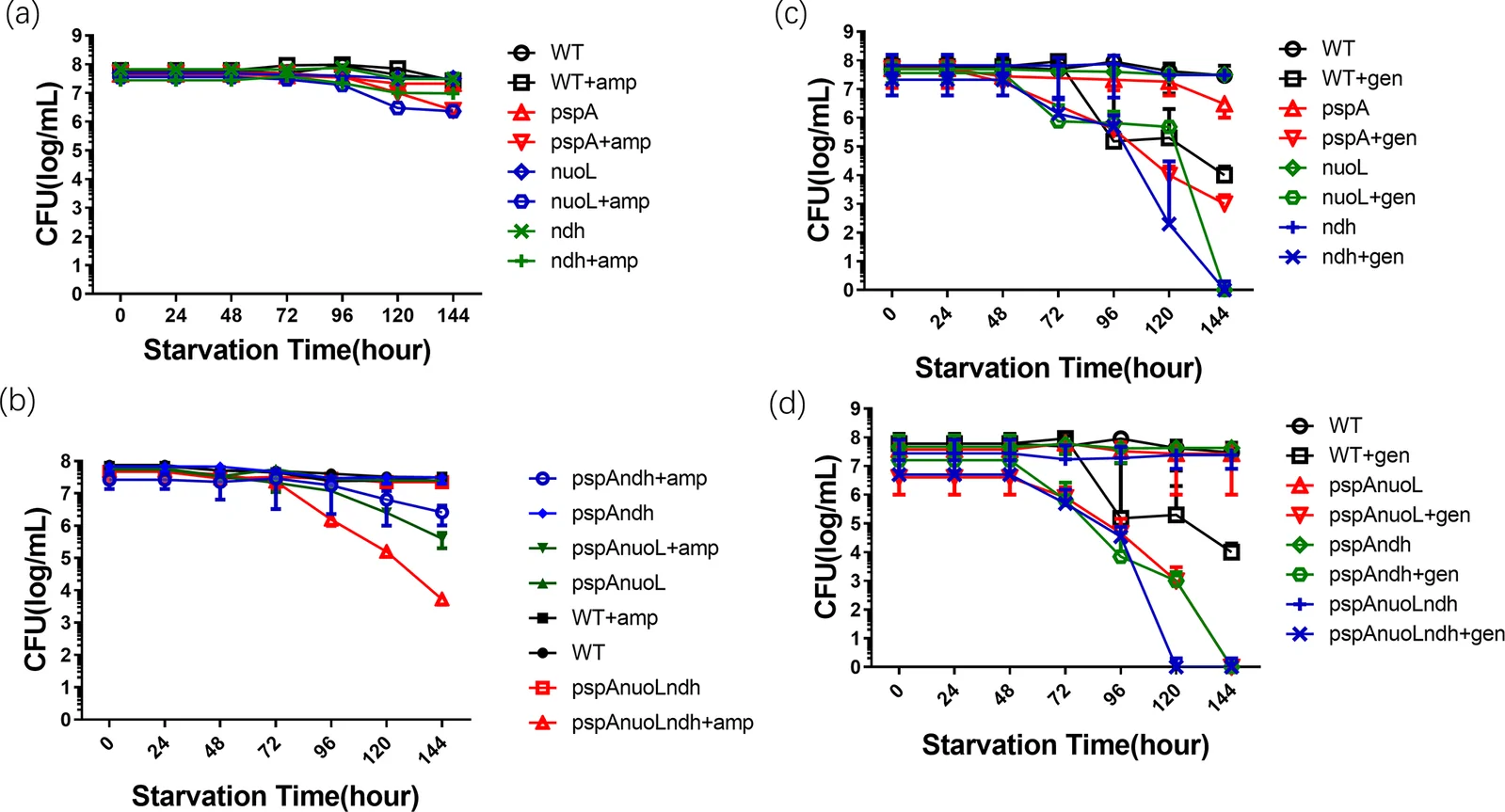
Tolerance level of knockout strains in which PMF maintenance and ETC genes were simultaneously deleted during a 6-day starvation experiment. Tolerance level of *pspA, nuoL,* and *ndh* (**a**) and *pspAnuoL, pspAndh*, and *pspAnuoLndh* (**b**) knockout strains with or without 100 µg/mL ampicillin treatment. Tolerance level of *pspA, nuoL,* and *ndh* (**c**) and *pspAnuoL, pspAndh*, and *pspAnuoLndh* (**d**) knockout strains with or without treatment with 10 µg/mL gentamicin. *E. coli* strain. BW25113 was included as wild-type control.

As the most functionally important genes were found to be *pspA*, *nuoL,* and *ndh*, which play a role in PMF maintenance and active generation of PMF, respectively, we, therefore, further tested the effect of simultaneous deletion of these genes. Yet, we found that the combined effect of deletion of genes in these two groups was not as strong as expected. In ampicillin treatment, the population size of persisters of the two double-knockout strains decreased to 10^7^/mL and 10^6^/mL only upon starvation for 6 days, while that of the *pspAnuoLndh* triple-knockout strain decreased to 10^4^, which is the same as that of the *nuoLndh* double-knockout strains (Fig. 4a and b). In gentamicin treatment, persisters of the *pspAnuoL* and *pspAndh* deletion mutants were eradicated by day 6, and those of the *pspAnuoLndh* triple-knockout strains were completely killed by day 5; this killing rate was the same as that observed for the *nuoLndh* double-knockout mutant (Fig. S2c and d).

### Membrane potential of gene knockout mutants

**Fig 5:**
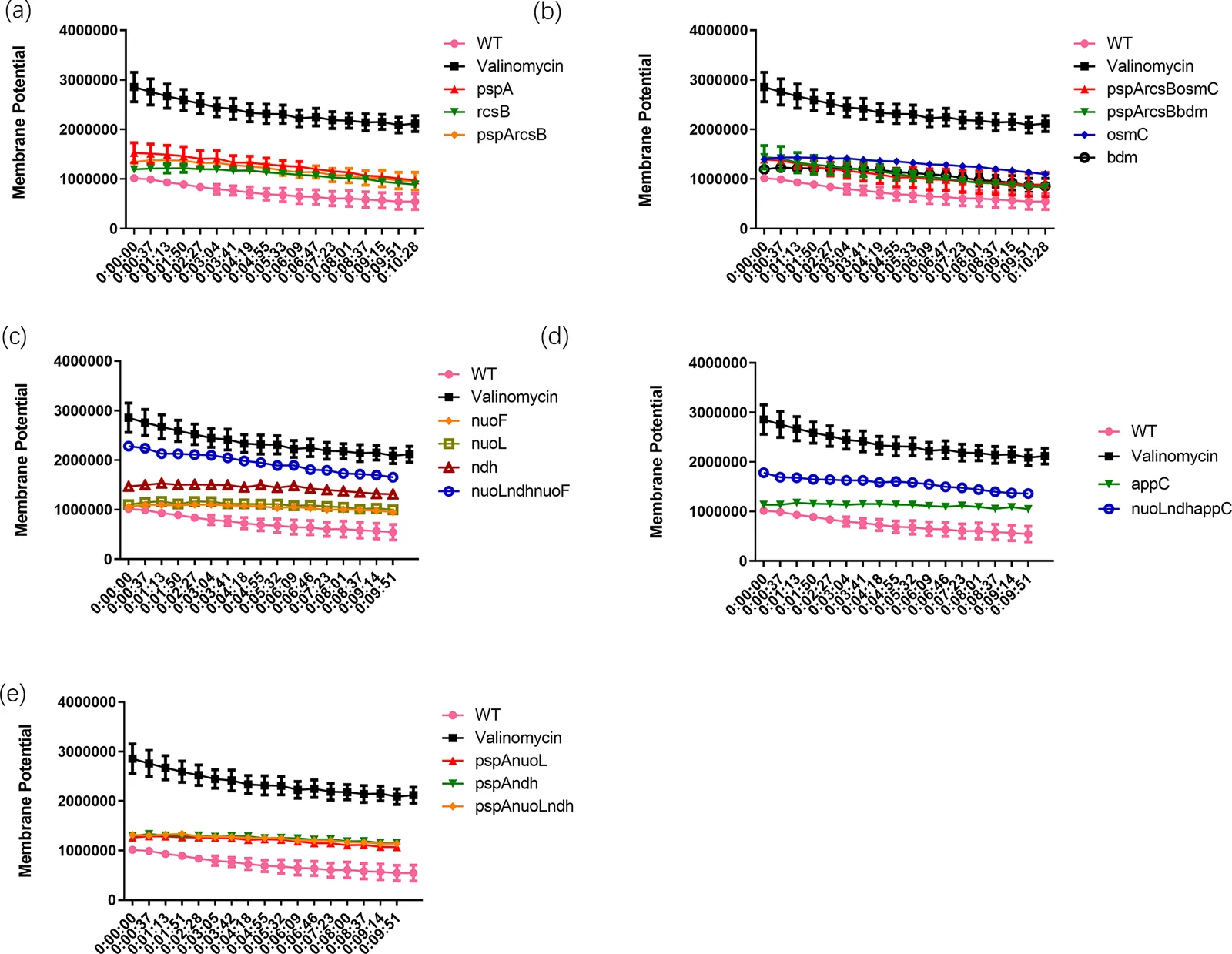
Membrane potential of various gene knockout strains. Membrane potential of *pspA, rcsB*, and *pspArcsB* (**a**); *osmC, bdm*, *pspArcsBosmC,* and *pspArcsBbdm* (**b**); *nuoF, nuoL, ndh,* and *nuoLndhnuoF* (**c**); *appC* and *nuoLndhappC* (**d**); and *pspAnuoL*, *pspAndh*, and *pspAnuoLndh* (e) knockout strains. A WT control was included. Valinomycin was included as control.

To investigate the mechanism underlying tolerance formation, we tested the membrane potential of strains in which the genes involved in maintaining and generating proton motive force were deleted. The membrane potential is normally created by establishing a proton (H+) gradient across the cell membrane. To prove that the Psp response and Rcs response can maintain PMF during starvation, we used the dye DiSC3(5) to test the degree of changes in bacterial cell membrane potential upon entry into the starvation mode. A significant level of accumulation of the dye in the bacteria cells would result in quenching of the overall fluorescence level of the cell suspension, whereas a rapid release of the dye into the medium would result in dequenching upon depolarization of the dye and increase in fluorescence signal. Figure 5 showed that the membrane potential of all the knockout strains of the *psp* and *rcs* operon was slightly higher than the wild type. Valinomycin, a depsipeptide antibiotic which can cause complete dissipation of the membrane potential (∆*ψ*), was included as control. The *psp* and *rcs* gene knockout strains were found to exhibit a significant increase in fluorescence signal, indicating a collapse in membrane potential; we, therefore, conclude that the PspA and Rcs response can contribute to tolerance formation through maintaining a high PMF. Likewise, the ETC gene knockout strains also exhibited a significant degree of dissipation of membrane potential, and the *nuoLndhappC* as well as *nuoLndhuoF* gene knockout strains exhibited almost the same level of fluorescence as that of the valinomycin control, suggesting that these four ETC genes play the role of generating PMF in persisters and, hence, maintaining the tolerance phenotype.

### Antibiotic accumulation in gene knockout strains

**Fig 6:**
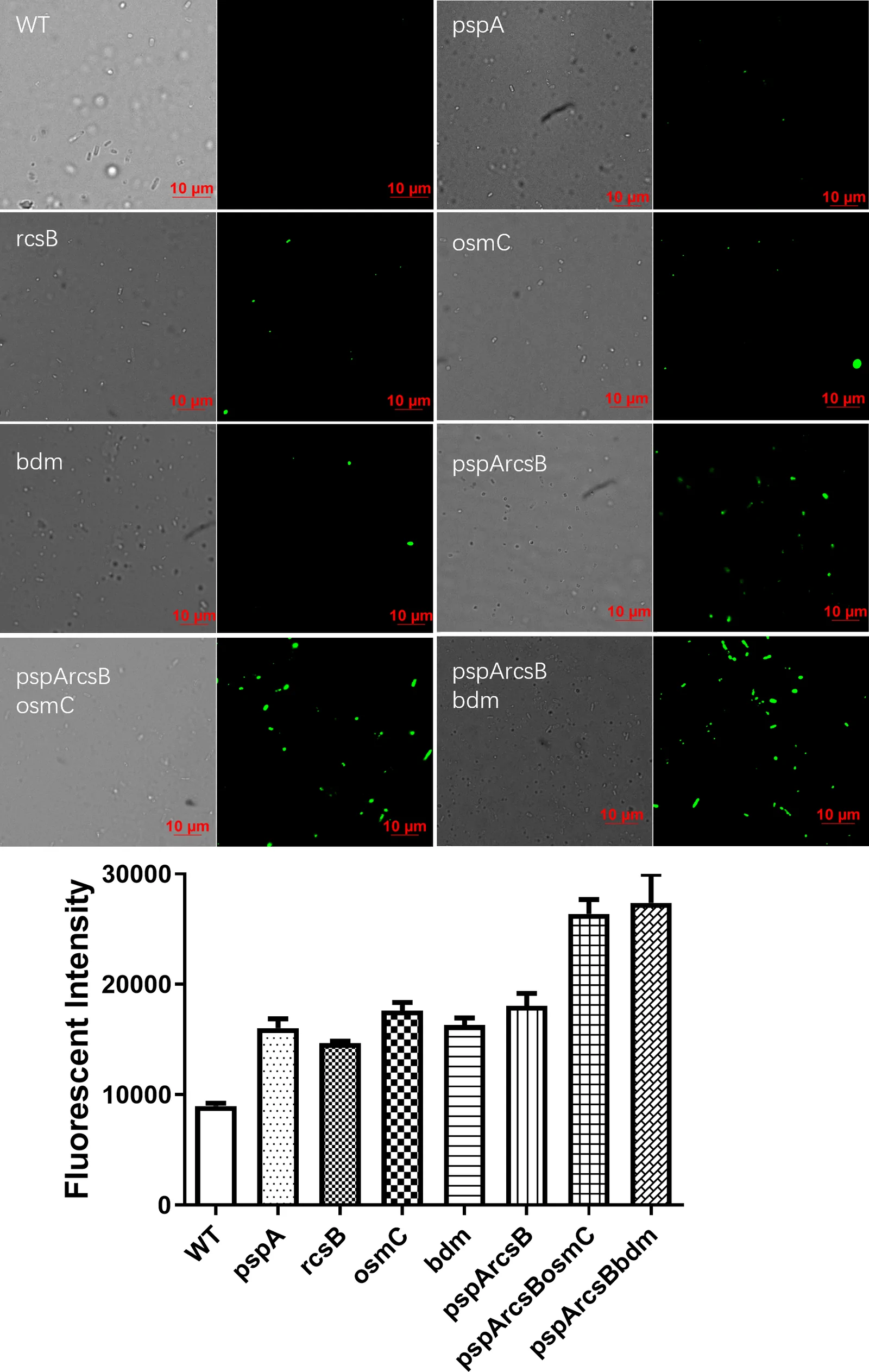
Ampicillin accumulation in strains in which specific PMF maintenance gene is deleted, with the wild-type strain as control. Top panel, image of fluorescence microscopy of bacterial cells subjected to assessment of the degree of accumulation of Bocillin FL. Lower panel, the degree of accumulation of Bocillin FL in different gene deletion mutants.

**Fig 7:**
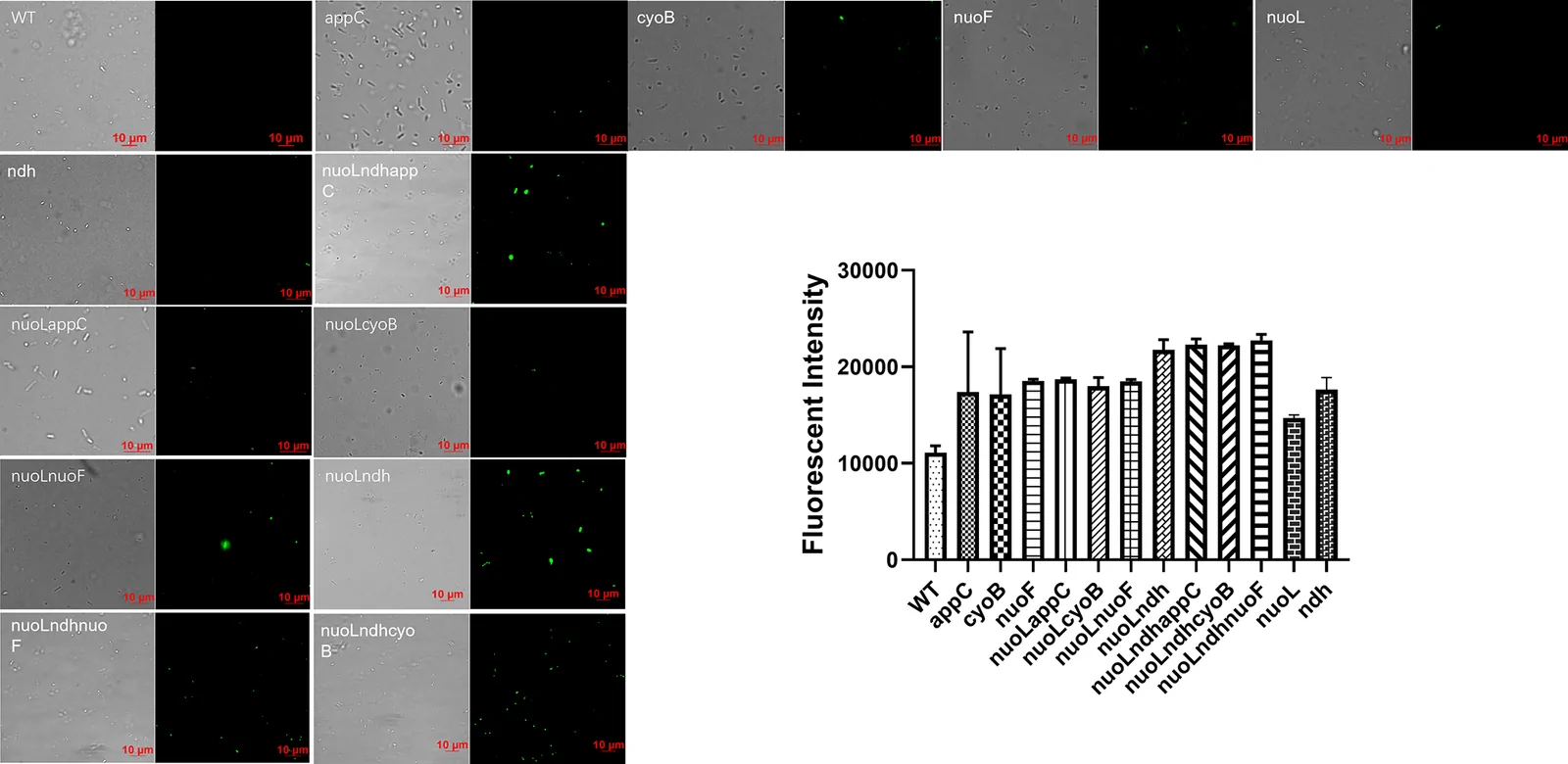
Ampicillin accumulation in ETC gene knockout strains, with the wild-type strain as control. Top panel, image of fluorescence microscopy of bacterial cells subjected to assessment of the degree of accumulation of Bocillin FL. Lower panel, the degree of accumulation of Bocillin FL in different gene deletion mutants.

**Fig 8:**
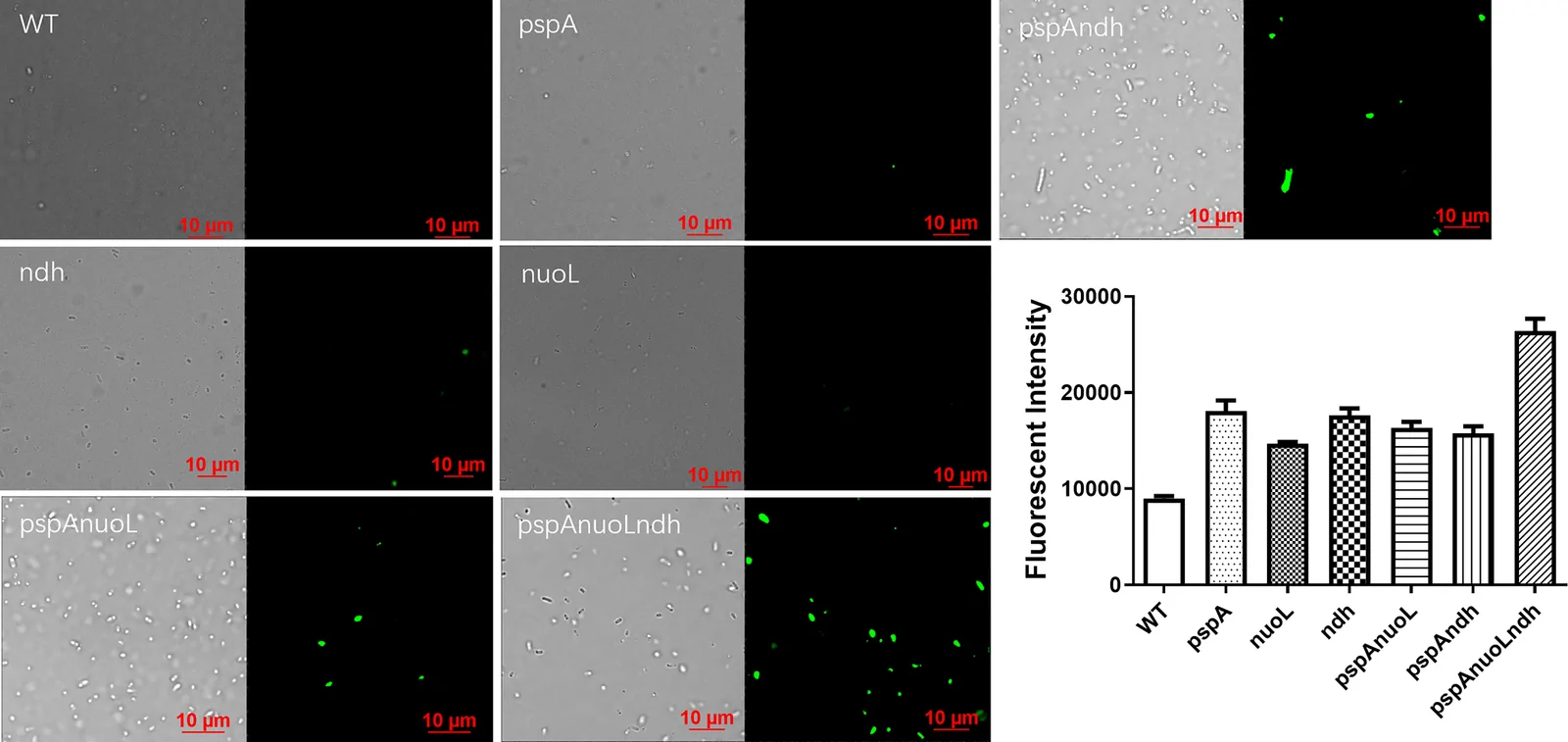
Ampicillin accumulation in which different combinations of PMF maintenance and ETC genes were deleted, with the wild-type strain as control. Top panel, image of fluorescence microscopy of bacterial cells subjected to assessment of the degree of accumulation of Bocillin FL. Lower panel, the degree of accumulation of Bocillin FL in different gene deletion mutants.

Specific efflux/transportation proteins were found to be able to pump β-lactam antibiotics out and mediate changes in PMF to enhance the tolerance level of *E. coli* BW25113 (27). In this work, we performed antibiotic accumulation assay to test the effect of deletion of genes involved in maintaining and generation of PMF. The degree of antibiotic accumulation in specific gene knockout strains subjected to starvation for 6 days was measured upon treatment with Bocillin FL penicillin. As shown in Fig. 6 to 8, Bocillin was almost undetectable in the wild-type strain, but significant accumulation could be detected in the *pspA, rcsB, osmC, bdm, nuoL,* and *ndh* gene knockout strains.

**Fig 9:**
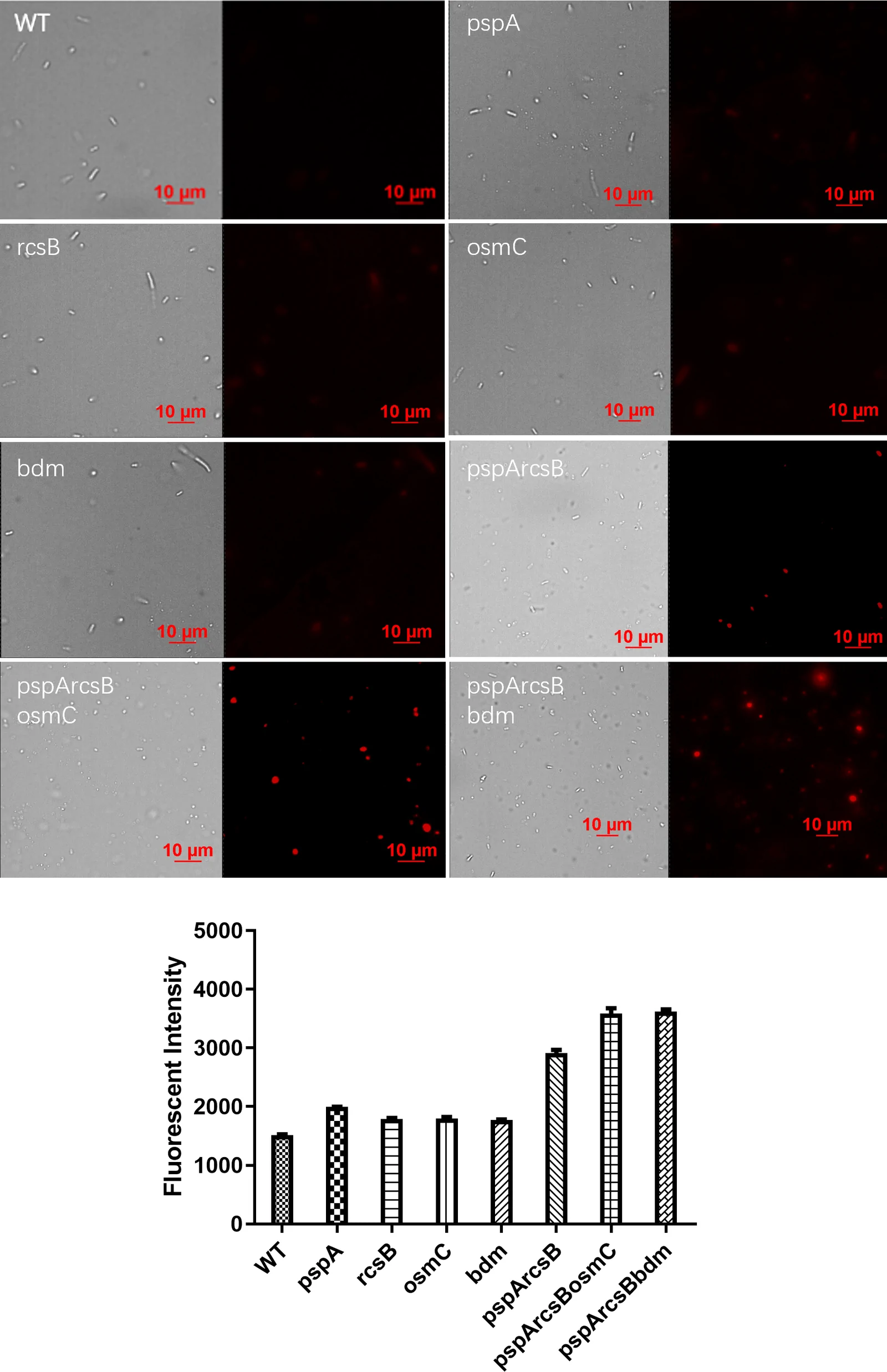
Gentamicin accumulation in strains in which specific PMF maintenance gene is deleted, with the wild-type strain as control. Top panel, image of fluorescence microscopy of bacterial cells subjected to assessment of the degree of accumulation of Texas red gentamicin conjugate. Lower panel, the degree of accumulation of Bocillin FL in different gene deletion mutants.

**Fig 10:**
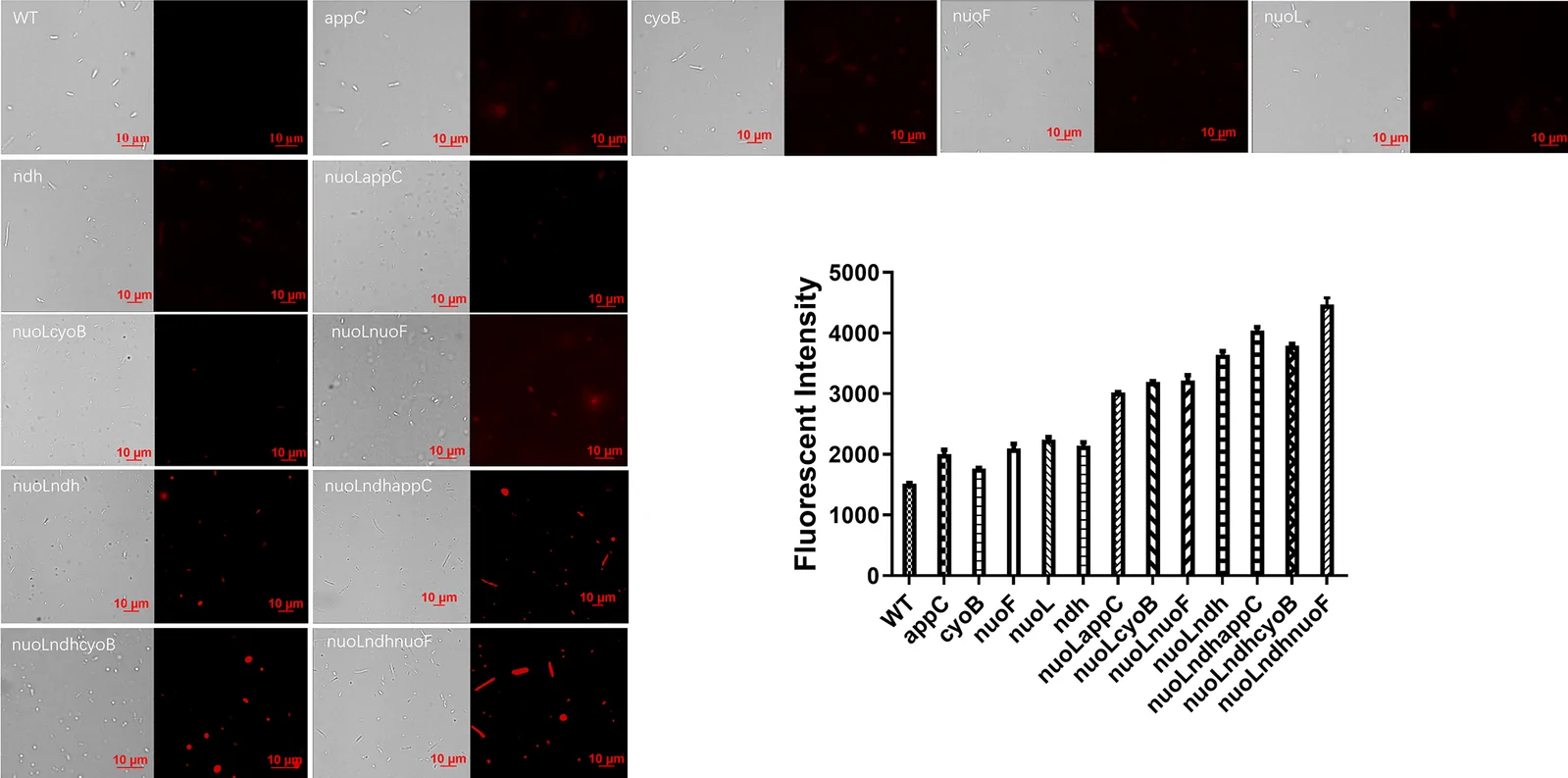
Gentamicin accumulation in ETC gene knockout strains, with the wild-type strain as control. Top panel, image of fluorescence microscopy of bacterial cells subjected to assessment of the degree of accumulation of Texas red gentamicin conjugate. Lower panel, the degree of accumulation of Bocillin FL in different gene deletion mutants.

**Fig 11:**
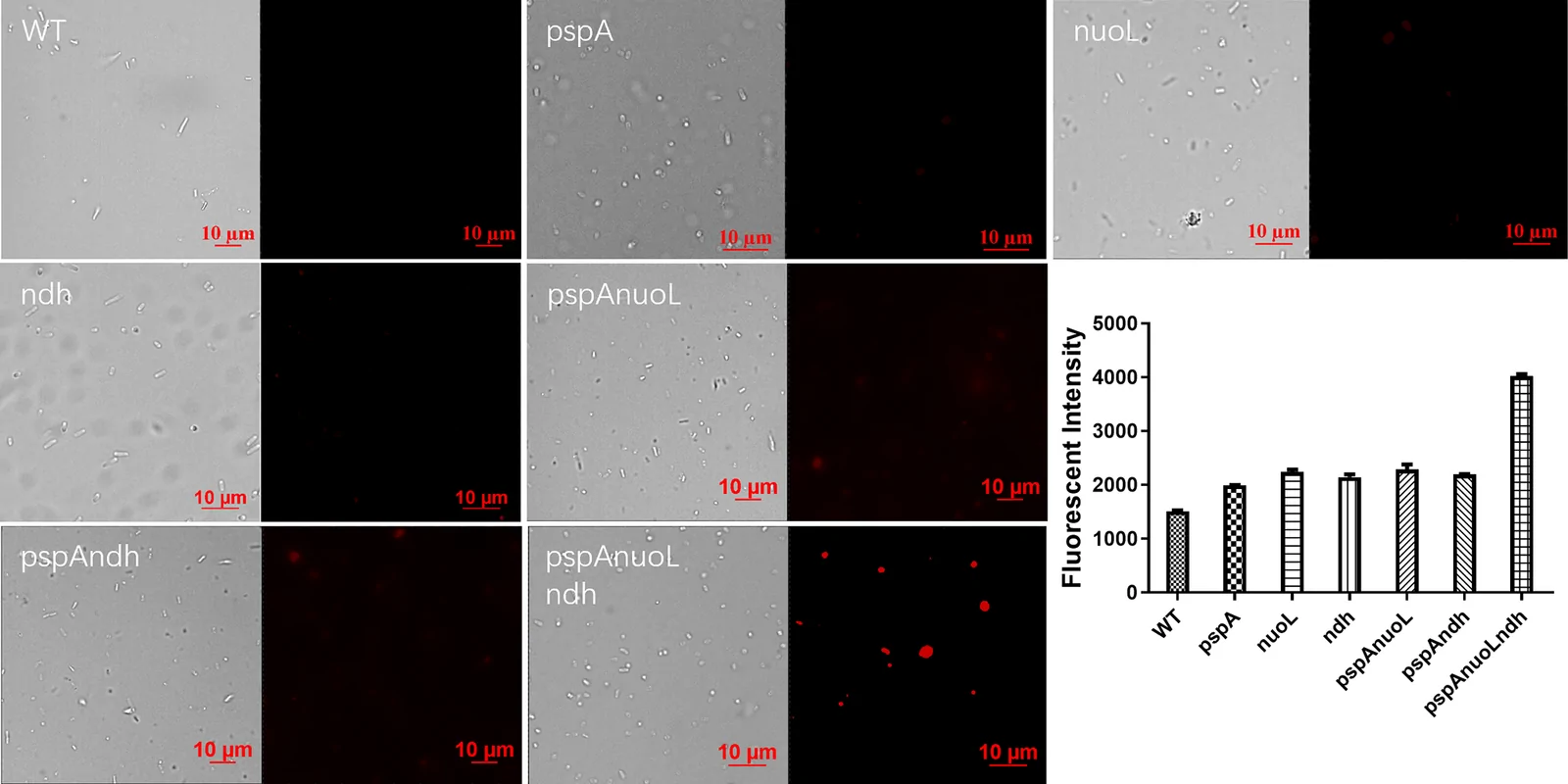
Gentamicin accumulation in which different combinations of PMF maintenance and ETC genes were deleted, with the wild-type strain as control. Top panel, image of fluorescence microscopy of bacterial cells subjected to assessment of the degree of accumulation of Texas red gentamicin conjugate. Lower panel, the degree of accumulation of Bocillin FL in different gene deletion mutants.

The double- and triple-gene knockout strains were also subjected to assessment of changes in the degree of antibiotic accumulation upon loss of functions in PMF generation and maintenance. The number of organisms which exhibited accumulation of Bocillin FL penicillin was much higher in the *pspArcsB*, *nuoLndh*, *pspArcsBosmC, pspArcsBbdm, nuoLndhappC*, *nuoLndhnuoF*, and *pspAnuoLndh* double- and triple-gene knockout strains when compared to the single- and double-knockout strains (Fig. 6 to 8). Likewise, gentamicin accumulation was also observed in those knockout strains (Fig. 9 to 11). In particular, a significantly larger amount of the Texas red gentamicin conjugate accumulated in the *pspArcsB*, *nuoLndh*, *pspArcsBosmC, pspArcsBbdm, nuoLndhappC*, *nuoLndhnuoF*, and *pspAnuoLndh* double- and triple-gene knockout strains when compared to the wild-type strain. Findings of the antibiotic accumulation experiments are, therefore, consistent with those of the tolerance assays and assessment of the membrane potential of gene deletion mutants in that dissipation of PMF strongly affects the functions of specific transporters that play an active role in the maintenance of phenotypic antibiotic tolerance in bacteria.

## DISCUSSION

Our previous study showed that a range of bacterial membrane proteins was actively expressed in antibiotic-tolerant persisters during starvation (18). A notable example is PspA, which plays a role in the maintenance of PMF and contributes to the expression of antibiotic tolerance. Based on this finding, we strived to identify other active tolerance responses involved in the maintenance of PMF. We found that the *rcsAB* system is also responsible for maintaining PMF in a manner similar to that of PspA. As a regulator of 19 proteins, deletion of several of the genes in this operon, especially the *osmC* gene which encodes an osmotically induced lipoprotein, and the *bdm* gene which encodes a periplasmic peroxidase, resulted in a significant reduction in antibiotic tolerance level during prolonged starvation. Interestingly, although deletion of both *pspA* and *rcsB* can lead to even lower tolerance level, the *pspAosmC* and *pspAbdm* double-knockout strains did not exhibit such effect. This observation can be explained by the fact that RcsB is a regulator of OsmC and Bdm; hence, the function of both these two genes was abolished in the *pspArcsB* double-knockout mutant, resulting in a lower tolerance level. This finding, therefore, indicates that PMF maintenance is an important mechanism underlying the onset of starvation-induced bacterial tolerance.

On the other hand, our finding confirms that the ability to generate PMF also contributed significantly to tolerance formation and maintenance. NuoL and Ndh are two major enzymes of the electron transport chain, namely, NADH dehydrogenase I and NADH dehydrogenase II, respectively. Importantly, deletion of the *nuoL* and *ndh* gene alone could cause a reduction in tolerance level in the same manner as that of deletion of *pspA*. A mutant in which the *pspA, nuoL,* and *ndh* genes were deleted exhibited a significantly decreased tolerance level under long-term starvation; likewise, deletion of both *pspA* and *nuoL* was also sufficient to cause a larger reduction in tolerance level when compared to deletion of *pspA* or *nuoL* alone, confirming that maintaining and generating PMF are both important mechanisms underlying expression of phenotypic antibiotic tolerance. In fact, our data appear to suggest that active generation of PMF is arguably more important than PMF maintenance in persisters if sustainable expression of tolerance phenotypes is required, as deletion of genes in the electron transport chain resulted in lower tolerance level than mutants in which PMF maintenance genes were deleted.

Measurement of the membrane potential of the single-, double-, and triple-knockout strains showed that they all exhibited significant reduction in membrane potential. Nevertheless, the *pspArcsB* double-knockout strain and the *pspArcsBosmC*, *pspArcsBbdm*, and *pspAnuoLndh* triple-knockout strains did not exhibit complete depletion of PMF. It is likely that there are still other genes in bacteria that are involved in the maintenance of PMF so that deletion of the PMF maintenance genes and genes encoding electron transport chain components was still not able to cause a total collapse of the membrane potential. Consistent with our previous finding is that a range efflux pumps were also involved in tolerance formation by playing a role in generating PMF (18), the antibiotic accumulation assay results indicated that expression of the PMF maintenance genes results in reduced antibiotic accumulation, presumably by supporting the functions of specific efflux pumps and enabling persisters to survive against long-term starvation. In our experiment, the number of bacteria which exhibited accumulation of Bocillin FL penicillin and the Texas red gentamicin conjugate was much higher in the *pspArcsBosmC, pspArcsBbdm, pspAnuoLndh* triple-knockout strains when compared to the single- and double-knockout strains. The result was consistent with the tolerance assay and results of assessment of membrane potential of the gene deletion mutants, confirming that PMF maintenance is an important tolerance mechanism that protects persisters against long-term starvation. In particular, our data showed that, although uptake of aminoglycoside is PMF-dependent, inhibition of the ability to maintain PMF in persisters actually results in a reduction in efflux activities and, hence, accumulation of aminoglycosides in the bacterial cells and eventually cell death. All in all, findings in this work showed that proteins responsible for maintaining and generating PMF are meaningful targets for the development of novel strategies to prevent and control recurrent and chronic infections.

## SUPPLEMENTAL MATERIAL

Supplemental figures spectrum.00832-23-s0001.pdf Figure S1 and Figure S2.

ASM does not own the copyrights to Supplemental Material that may be linked to, or accessed through, an article. The authors have granted ASM a non-exclusive, world-wide license to publish the Supplemental Material files. Please contact the corresponding author directly for reuse.
