# Supplementary material for: Maintenance and generation of proton motive force are both essential for expression of phenotypic antibiotic tolerance in bacteria
Source: Microbiol Spectr. 2023 Aug 25;11(5):e00832-23. doi: 10.1128/spectrum.00832-23 (PMC10580908; doi:10.1128/spectrum.00832-23)
Supplement: Supplemental figures — Figure S1 and Figure S2. [file spectrum.00832-23-s0001.pdf]

Supplementary

Maintenance and generation of proton motive force are both essential for expression of phenotypic antibiotic tolerance in bacteria

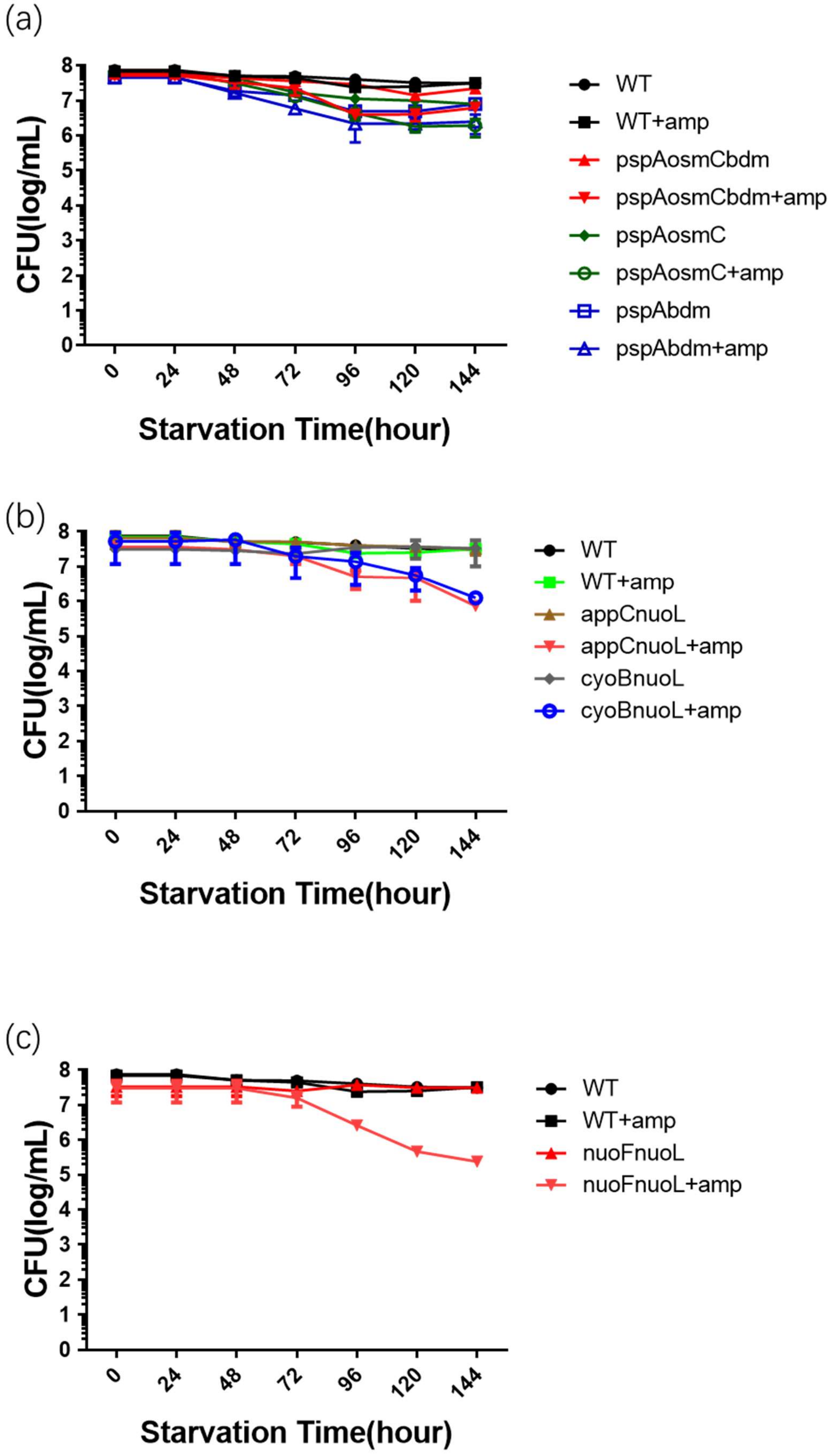

Figure S1. Level of tolerance to ampicillin in ETC and PMF maintenance gene

knockout strains under 6-day-starvation. Tolerance level of *pspAosmC, pspAbdm, pspAosmCbdm* (a), *appCnuoL, cyoBnuoL* (b)*, nuoFnuoL* (c) gene knockout strains with or without 100 µg/ml ampicillin treatment. *E. coli* strain. BW25113 was included as wild type control.

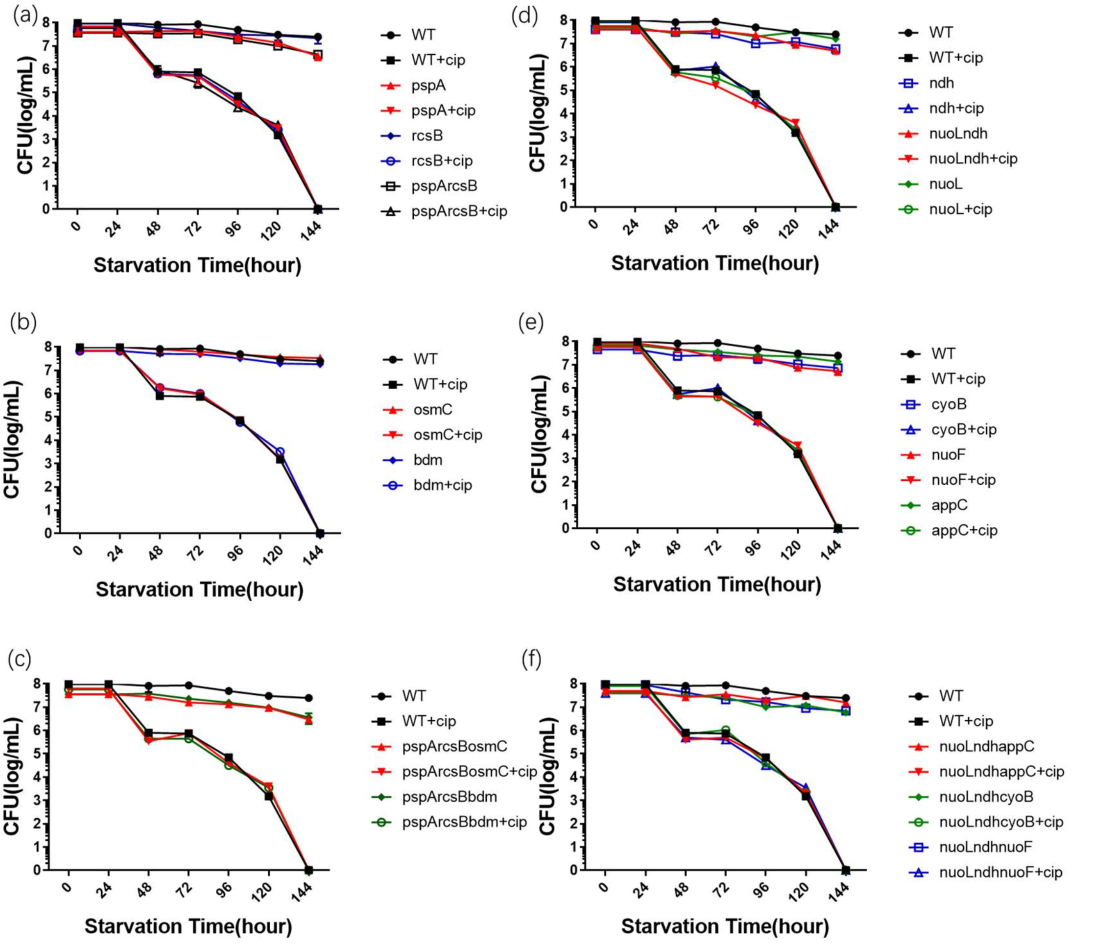

Figure S2. Changes in level of tolerance of ETC and PMF maintenance gene knockout strains to ciprofloxacin during the course of 6-days starvation. Tolerance level of *pspA, rcsB, pspArcsB* (a), *osmC, bdm* (b)*, pspArcsBosmC, pspArcsBbdm* (c), *nuoL, ndh, nuoLndh* (d), *cyoB, appC,* (e)*, nuoLndhappC, nuoLndhcyoB, nuoLndhnuoF* (f) knockout strains with or without treatment by 4 g/ml ciprofloxacin. *E. coli* strain. BW25113 was included as wild type control.
